# Knowledge and attitude of pregnant women towards preeclampsia and its associated factors in South Gondar Zone, Northwest Ethiopia: a multi‐center facility‐based cross‐sectional study

**DOI:** 10.1186/s12884-021-03647-2

**Published:** 2021-02-23

**Authors:** Maru Mekie, Dagne Addisu, Minale Bezie, Abenezer Melkie, Dejen Getaneh, Wubet Alebachew Bayih, Wubet Taklual

**Affiliations:** 1 Department of Midwifery, College of Health Sciences, Debre Tabor University, Debre Tabor, Ethiopia; 2 Department of Pediatrics and Child Health Nursing, College of Health Sciences, Debre Tabor University, Debre Tabor, Ethiopia; 3 Department of Public Health, College of Health Sciences, Debre Tabor University, Debre Tabor, Ethiopia

**Keywords:** Preeclampsia, Knowledge, Attitude, Maternal mortality, Ethiopia

## Abstract

**Background:**

Preeclampsia has the greatest impact on maternal mortality which complicates nearly a tenth of pregnancies worldwide. It is one of the top five maternal mortality causes and responsible for 16 % of direct maternal death in Ethiopia. Little is known about the level of knowledge and attitude towards preeclampsia in Ethiopia. This study was designed to assess the knowledge and attitude towards preeclampsia and its associated factors in South Gondar, Northwest Ethiopia.

**Methods:**

A multicenter facility-based cross-sectional study was implemented in four selected hospitals of South Gondar Zone among 423 pregnant women. Multistage random sampling and systematic random sampling techniques were used to select the study sites and the study participants respectively. Data were entered in EpiData version 3.1 while cleaned and analyzed by Statistical Package for Social Sciences (SPSS) version 23. Descriptive and inferential statistics were performed. Adjusted odds ratio with 95 % confidence interval were used to identify the significance of the association between the level of knowledge on preeclampsia and its predictors.

**Results:**

In this study, 118 (28.8 %), 120 (29.3 %) of the study participants had good knowledge and a positive attitude towards preeclampsia respectively. The likelihood of having good knowledge on preeclampsia was found to be low among women with no education (AOR = 0.22, 95 % CI (0.06, 0.85)), one antenatal care visit (ANC) (AOR = 0.13, 95 % CI (0.03, 0.59)). Whereas, those who booked for ANC in the first trimester (AOR = 6.59, 95 % CI (1.43, 30.33)), gave the last birth at a health facility (AOR = 2.61, 955 CI (1.03, 6.61)), and experienced a complication during previous births (AOR = 3.67, 95 % CI (1.78, 7.57)) were more likely to be knowledgeable on preeclampsia.

**Conclusions:**

No formal education and not attending four ANC visits were associated with poor knowledge of preeclampsia. While participants who visited health facilities during the first trimester, who gave birth at health facilities, and those who experienced a complication in previous births were more likely to be knowledgeable on preeclampsia. Improving the numbers of ANC visits and encouraging facility delivery are important measures to improve women’s knowledge on preeclampsia. Health education regarding preeclampsia risk factors, symptoms, and complications shall be emphasized.

## Background

Preeclampsia has the greatest impact on maternal mortality which complicates nearly a tenth of pregnancies worldwide [1–4]. It is the second leading cause of direct maternal death and directly responsible for 70,000 maternal deaths annually at the global level [4, 5] although the exact prevalence of morbidity and mortality related to preeclampsia is not reported in the developing countries. Preeclampsia is one of the top five maternal mortality causes and responsible for 16 % of direct maternal death in Ethiopia [6–8]. The majority of deaths related to preeclampsia could be averted by evidence-based, effective, and timely interventions by increasing women’s knowledge and changing attitudes towards preeclampsia [9, 10].

Studies indicated that pregnant women have poor knowledge and wrong perception about preeclampsia despite its relevance for early identification and management of the problem [8, 11]. A study conducted in a District Hospital in Tanzania indicated that 60 % of the study participants did not know the consequences of preeclampsia. Lack of knowledge is found to be the predisposing factor to practice risky behaviors for preeclampsia. In the same way, poor understanding of the disease leads to anxiety and becomes a source of stress to the family as well [12, 13].

Globally, the maternal mortality ratio declined to 216 per 100, 000 live births from 385 to 100,000 live births in 2015 [14] despite the reduction was not consistent across countries. Countries have to strive hard to accomplish the Sustainable Development Goals (SDGs) which demands more commitment than Millennium Development Goals (MDGs) [15]. Morbidity and mortality related to preeclampsia might be attributed to poor knowledge and negative attitude of pregnant women.

There are several problems related to preeclampsia prevention and diagnosis such as challenges in the prediction, prevention, delay in transport, and management of preeclampsia which are complemented by the shortage of trained health personnel and poor health infrastructures in developing countries [11]. To address the burden of preeclampsia-related consequences, shifting from clinic-based care to community-based case-ascertainment and treatment are key intervention strategies especially in resource-limited areas [5]. Studies conducted in different parts of the world indicated that there is a significant gap among pregnant women related to knowledge, attitude, and perception towards preeclampsia [16–18] which directly or indirectly influence health seeking-behavior leading to increased maternal mortality and morbidity [12, 13].

A study in Australia revealed that 77 % of women did not know about preeclampsia before diagnosis. Even among women who were diagnosed with preeclampsia, half of them did not understand the seriousness of preeclampsia [16]. Understanding the level of knowledge and attitude of women about preeclampsia is essential to undertake appropriate measures [16, 19]. To the best of the authors’ knowledge, studies are limited in Ethiopia regarding the knowledgeand attitude of pregnant women towards preeclampsia.

## Methods

### Study Design and Period

A multicenter facility-based cross-sectional study design was implemented in four selected hospitals (Debre Tabor General Hospital, Addis Zemen Primary Hospital, Mekane Eyesus Primary Hospital, and Nefas Mewucha Primary Hospital found in South Gondar Zone of Northwest Ethiopia from January 5 to February 5/2020. The four hospitals were selected for the study purpose out of eight hospitals in the Administrative Zone.

### Study Population

All pregnant women who came to receive antenatal care (ANC) services in the selected Hospitals were the source population. While pregnant women who visit the ANC clinics in the selected Hospitals during the study period were the study population. On the other hand, those who visited the ANC clinics for the diagnosis of pregnancy were excluded.

### Sample size determination and Sampling procedures

A single population proportion formula was used to calculate sample size at 95 % confidence interval, 5 % margin of error, and taking proportion (p) of 0.5 (since therewas no similar previous study). Thus, a prevalence of 50 % was taken to obtain the maximum possible sample size. After adding a 10 % non-response rate, the final sample was 423. A multistage random sampling technique was used to select study participants by stratifying hospitals as primary and general hospitals. As of 2020, there are 8 hospitals in South Gondar Zone Administration (1 general and 7 primary hospitals). Debre Tabor General Hospital (DTGH) was taken for the general hospital. While, three primary hospitals; Addis Zemen Primary Hospital (AZPH), Mekane Eyesus Primary Hospital (MEPH), and Nefas Mewucha Primary Hospital (NMPH) were selected by lottery method. Then the calculated sample was proportionally allocated to the selected hospitals based on the ANC case load. The individual study participants were selected by systematic random sampling technique. The six-month caseload was assessed for the selected hospitals to proportionally allocate the sample. By converting the six-month caseload to four weeks (the proposed data collection period), sampling interval was identified. Hence, the four weeks ANC case load in DTGH, AZPH, MEPH, and NMPH were 1200, 360, 320, and 340 respectively. Therefore, the allocated samples were 229, 68, 61, and 65 for DTGH, AZPH, MEPH, and NMPH respectively. A sampling interval of 5 was used to select the study participants.

### Variables and Measurements

#### Knowledge

 The knowledge of the study participants was assessed using the modified Bloom’s cut-off point [20]. The knowledge of the study participants was categorized as good if the participants scored between 80 and 100 % (12–15 points), moderate if scored between 50 and 79 % (7.5–11 points), and poor if they scored less than 50 % (< 7.5 points). For the sake of data management, those who had a score of 80–100 % and those with a score of < 80 % were reported as having good knowledge and not having good knowledge (moderate and poor knowledge) respectively.

#### Attitude

 Attitude was reported as positive, neutral and negative when they scored 80–100 %, 60–79 %, and less than 60 % of the attitude assessment questions respectively using the Bloom’s cut-off point [20].

### Data Collection Procedures and Quality Assurance

Data were collected using a structured questionnaire through face to face interview. The questionnaire was adapted from different published literature [19, 21, 22]. A pretest of the questionnaire was conducted on 22 pregnant women outside the study area to undertake necessary amendments. The number of participants included in the pretest was 5 % of the number of pregnant women calculated as the sample size. The data collection tool has four sections. These are socio-demographic parts, obstetric characteristics, knowledge, and attitude measurement questions related to preeclampsia. The questionnaire was prepared in English, translated to Amharic, and then back to English to ensure consistency. To ascertain data quality, data collection and supervision were facilitated by trained health professionals. Training was given for data collectors and supervisors for two days duration to minimize measurement bias. Data collected for pretesting was not included in the final report of the research.

### Data Management and Analysis

Data were entered in a template prepared in EpiData version 3.1 and exported to SPSS version 23 data cleaning, editing, and analysis. Descriptive statistics such as frequency and percentage were used to describe the characteristics of the study participants.

Variables with a P-value of ≤ 0.2 in bivariable logistic regression were entered in the multivariable model to identify factors associated with the level of knowledge of pregnant women towards preeclampsia. Variance Inflation Factor (VIF) was used to check the possible multicollinearity between independent variables. VIF of ≥ 10 was used as cutoff point for the presence of multicollinearity between independent variables. The Hosmer-Lemeshow goodness of fit test was used to assess the fitness of the multivariable model. The Hosmer-Lemeshow goodness of fit test indicated that the multivariable model was fit at X^2^ = 12.95 with P-value of 0.114. In the multivariable model, a P-value of ≤ 0.05 was used to decide statistical significance at a 95 % confidence level.

### Ethical Consideration

 Ethical clearance was obtained from the research ethics committee of the College of Health Sciences in Debre Tabor University. Written consent was obtained from each study participant after a detailed description of the study aims. The privacy of respondents and confidentiality of information was ensured.

## Results

### Socio‐demographic characteristics of the Study participants

In this study, 410 women who visited the selected hospitals for ANC visits were completed the interview giving a response rate of 96.93 %. The mean and the median age of the study participants were found to be 28.42 and 28 years respectively with a range of 17 to 45 years. A high proportion, 334 (81.5 %) of the study participants were found in age ranges of 17–34 years while 76 (18.5 %) were found in age groups of 35–45 years.

A High proportion of the study participants were Urban residents 277 (67.6 %), in union 391 (95.4 %), and housewives 167 (40.7 %). The median monthly household income of the study participants was 3000 ETB (United States Dollar (USD) 94.37$) with a minimum and maximum income of 400 ETB (USD 12.58$) and 20,000 ETB (USD 629.13$) respectively (Table 1).

**Table 1:** Socio-demographic characteristics of pregnant women in selected Hospitals of South Gondar Zone, Ethiopia, 2020

Variables	Frequency (N)	Percent (%)	Chi-square (X^2^)	*P*-value
**Age in years**				
17–34	334	81.5	0.356	0.55
35–45	76	18.5		
**Residence**				
Urban	277	67.6	6.906	0.009
Rural	133	32.4		
**Marital status**				
In union	391	95.4	1.726	0.19
Not in union	19	4.6		
**Education level**				
No formal education	131	32.0	23.785	< 0.001
Primary education	102	24.9		
Secondary education	103	25.1		
College and above	74	18.0		
**Occupation**				
Housewife	167	40.7	19.918	0.001
Merchant	100	24.4		
Daily laborer	13	3.2		
Farmer	62	15.1		
Government employee	68	16.6		
**Monthly household income**				
< 3000 ETB (94.37$)	180	43.9	6.733	0.009
≥ 3000 ETB (94.37$)	230	56.1		

*NB* Average ETB, USD $ exchange rate (1 $ = 31.79 ETB)

### Obstetrics and medical condition of the study participants

With regards to the numbers of births, 88 (21.5 %) and 30 (7.3 %) of the study participants were primiparous (para 1) and grand multiparous (para 5 and above) respectively. Among participants who had a history of previous births, 179 (68.06 %) have reported as they gave birth at health facilities while 84 (31.94 %) reported as they gave birth at home. More than a quarter, 70 (26.62 %) of the study participants reported that they have experienced complications in their previous births.

Concerning the family history of a medical condition, 20 (4.9 %) and 8 (2 %) had a family history of chronic hypertension and diabetes mellitus respectively (Table 2).

**Table 2:** Obstetrics and Medical characteristics of pregnant women in selected Hospitals of South Gondar Zone, Ethiopia, 2020

Variables	Frequency (N)	Percent (%)	Chi-square (X^2^)	P-value
**Age at first pregnancy**				
< 18 years	30	7.3	1.217	0.27
≥ 18 years	380	92.7		
**Gravidity**				
1	147	35.9	0.831	0.660
2–4	227	55.4		
≥ 5	36	8.8		
**Parity**				
1	88	21.5	1.205	0.547
2–4	145	35.4		
≥ 5	30	7.3		
**Gestational age at first ANC visit**				
First trimester	88	21.5	5.170	0.075
Second trimester	170	41.5		
Third trimester	152	37.1		
**Numbers of ANC visits**				
1 visit	150	36.6	15.453	< 0.001
2–3 visits	165	40.2		
≥ 4 visits	95	23.2		
**Last place of birth**				
Health facility	179	68.06	21.841	< 0.001
Home	84	31.94		
**An obstetric complication in the previous births**				
Yes	70	26.62	18.819	< 0.001
No	193	73.38		
**Family history of HTN**				
Yes	20	4.9	4.619	0.032
No	390	95.1		
**Family history of DM**				
Yes	8	2.0	0.057	0.811
No	402	98.0		

### The knowledge and attitude of pregnant women towards preeclampsia

In this study, 92 (22.45 %), 200 (48.8 %), and 118 (28.8 %) of the study participants had poor, moderate, and good knowledge on preeclampsia respectively.

With regards to attitude towards preeclampsia, 26 (6.3 %), 264 (64.4 %), and 120 (29.3 %) of the study participants had negative, neutral, and positive attitudes towards the risk factors, the prevention, symptoms, and complications of preeclampsia. Most women agreed that early health-seeking 345 (84.2 %) and having regular ANC follow-up 340 (82.9 %) can reduce the complications related to preeclampsia. On the other hand, significant numbers of women were unsure whether reduced urine output 173 (42.2 %) and convulsion 139 (33.9 %) are related to preeclampsia or not (Table 3).

**Table 3:** Attitude towards preeclampsia among pregnant women in selected Hospitals of South Gondar Zone, Ethiopia, 2020

Attitude related questions	Agree N(%)	Unsure N(%)	Disagree N(%)
Avoiding stress reduces the risk of preeclampsia	261 (63.6)	22 (5.4)	127(31.0)
Having regular ANC is used to prevent preeclampsia	340(82.9)	47 (11.5)	23 (5.6)
Taking fruit and vegetable foods can reduce the risk of preeclampsia	262 (63.9)	56 (13.7)	92 (22.4)
Reducing high coffee consumption reduces the risk of preeclampsia	234 (57.1)	90 (22.0)	86 (21.0)
Avoiding alcohol consumption reduces the risk of preeclampsia	214 (52.2)	154 (37.6)	42 (10.2)
Preeclampsia is serious for the health of the mother	323 (78.80)	72 (17.6)	15 (3.7)
Preeclampsia is dangerous for the fetus in the womb	333 (81.2)	63 (15.4)	14 (3.4)
Early health-seeking related to preeclampsia reduces complication	345 (84.2)	52 (12.7)	13 (3.1)
Convulsion is the consequence of preeclampsia	252 (61.5)	139 (33.9)	19 (4.6)
Reduced urine output is related to the consequence of preeclampsia	107 (26.0)	173 (42.2)	130 (31.7)

### Factors associated with knowledge of preeclampsia among pregnant women

The education level of the study participants, gestational age at ANC booking, numbers of ANC visits, last place of birth, and experience of complications were found to be significantly associated with knowledge of preeclampsia in this study (Table 4).

**Table 4:** Multivariable analysis of factors associated with knowledge of preeclampsia among pregnant women in selected Hospitals of South Gondar Zone, Ethiopia, 2020

Variables	Have good Knowledge		COR with 95 % CI	AOR with 95 % CI
	**Yes N (%)**	**No N (%)**		
**Residence (*N* = 410)**				
Urban	91 (77.1)	186 (63.7)	1.92 (1.18, 3.14)^a^	1.21 (0.50, 2.91)
Rural	27 (22.9)	106 (36.3)	1	1
**Marital status (*N* = 410)**				
In union	110 (93.2)	281 (96.2)	0.54 (0.21, 1.37)	0.69 (0.17, 2.79)
Not in union	8 (6.8)	11 (3.8)	1	1
**The education level of the participants (*N* = 410)**				
No formal education	23 (19.5)	108 (37.0)	0.21 (0.11, 0.40)^a^	0.22 (0.06 ,0.85)^b^
Primary education	23 (19.5)	79 (27.1)	0.29 (0.15, 0.56)^a^	0.28 (0.07, 1.07)
Secondary education	35 (29.7)	68 (23.3)	0.52 (0.28, 0.95)^a^	0.79 (0.23, 2.68)
College and above	37 (31.4)	37 (12.7)	1	1
**Occupation of the participants (*N* = 410)**				
Housewife	46 (39.0)	134 (45.9)	0.41 (0.23, 0.73)^a^	1.76 (0.53, 5.85)
Merchant	33 (28.0)	67 (22.9)	0.59 (0.31, 1.11)	2.80 (0.77,10.20)
Farmer	8 (6.8)	54 (18.5)	0.18 (0.07,0.43)^a^	1.16 (0.24, 5.56)
Government employee	31 (26.3)	37 (12.7)	1	1
**Monthly household income (*N* = 410)**				
< 3000 ETB	40 (33.9)	140 (47.9)	0.56 (0.36, 0.87)^a^	1.46 (0.64, 3.34)
≥ 3000 ETB	78 (66.1)	152 (52.1)	1	1
**Gestational age at first ANC booking (*N* = 410)**				
First trimester	27 (22.9)	61 (20.9)	0.85 (0.49, 1.50)	6.59 (1.43, 30.33)^b^
Second trimester	39 (33.1)	131 (44.9)	0.57 (0.35, 0.94)^a^	0.64 (0.23, 1.73)
Third trimester	52 (44.1)	100 (34.2)	1	1
**Numbers of ANC visits (*N* = 410)**				
1 visit	32 (27.1)	118 (40.4)	0.34 (0.20, 0.60)^a^	0.13 (0.03, 0.59)^b^
2–3 visits	44 (37.3)	121 (41.4)	0.46 (0.27, 0.78)^a^	0.66 (024, 1.80)
≥ 4 visits	42 (35.6)	53 (18.2)	1	1
**Last place of birth (*N* = 263)**				
Health facility	67 (89.3)	112 (59.6)	5.68(2.58,12.51)^a^	2.61 (1.03,6.61)^b^
Home	8 (10.7)	76 (40.4)	1	1
**An obstetric complication in the previous births (*N* = 263)**				
Yes	34 (45.3)	36 (19.1)	3.50 (1.96, 6.27)^a^	3.67 (1.78, 7.57)^b^
No	41 (54.7)	152 (80.9)	1	1
**Family history of HTN (*N* = 410)**				
Yes	10 (8.5)	10 (3.4)	2.61 (1.06, 6.45)^a^	2.66 (0.71, 9.95)
No	108 (91.5)	282 (96.6)	1	1

Key: *AOR *Adjusted Odds Ratio, *COR* Crude Odds Ratio, ^a^ significant in the bivariable model, ^b^ significant in the multivariable model

**NB**: *In union refers to being married or living together while not in union refers to single, divorced, or widowed.*

The odds of having good knowledge on preeclampsia were found to be low among women who did not attend any formal education compared with those who attended college and above level of education (AOR = 0.22, 95 % CI (0.06, 0.85)). Women who visited health facilities for ANC booking in the first trimester were more likely to have good knowledge on preeclampsia compared to those who visited health facilities for ANC booking in the third trimester of pregnancy (AOR = 6.59, 95 % CI (1.43, 30.33)). Similarly, the number of ANC visits was found to be the predictor of having good knowledge of preeclampsia. Women who had only one ANC visit were less likely to have good knowledge on preeclampsia compared with those who attended four and more ANC visits (AOR = 0.13, 95 % CI (0.03, 0.59)).

With regards to the last place of birth, women who gave birth at health facilities in their last birth were 2.61 times more likely to have good knowledge on preeclampsia compared with their counterparts (AOR = 2.61, 955 CI (1.03, 6.61)). Likewise, participants who experienced obstetric complications in their previous births were 3.67 times more likely to have good knowledge of preeclampsia compared with counterparts (AOR = 3.67, 95 % CI (1.78, 7.57)). However, a statistically significant association was not found between the place of residence, occupation of the women, monthly household income, and family history of hypertension in the multivariable model while controlling for confounding factors.

## Discussion

Preeclampsia has a high case fatality rate compared with other pregnancy-induced hypertensive disorders. Hence, aside from diagnosing and treating patients who seek treatment, assessing the level of knowledge and attitude towards preeclampsia is relevant to design appropriate intervention mechanisms for the community [6, 23]. The purpose of this study was to assess the level of knowledge and attitude of pregnant women towards preeclampsia and its associated factors in the selected Hospitals of South Gondar Zone. The education status of the participants, gestational age at the first ANC booking, numbers of ANC visits, last place of birth, and obstetric complications in previous births were found to be significant predictors of having knowledge of preeclampsia.

Similar to a previous study [24], the level of good knowledge on preeclampsia among women who receive ANC in selected hospitals of South Gondar Zone was 118 (28.8 %). The finding of our study is higher than a study conducted in Ghana [25]. But lower than a study conducted at Hospitals of northern Ethiopia which was conducted about pregnancy-induced hypertension [26]. The discrepancy might be related to the variations between the scopes of the study in which the aforementioned study was conducted about pregnancy-induced hypertension in general in contrast to our study which was about preeclampsia. Similarly, the variation in the measurement of the outcome variable might be the reason for the discrepancy. The Bloom’s knowledge classification was used in our study in contrast to the study in northern Ethiopia which used the mean knowledge score.

A significant difference in the level of knowledge was observed across different signs and symptoms of preeclampsia which varies from 383 (93.4 %) high blood pressure and 92 (22.4 %) genital bleeding as a symptom of preeclampsia. The finding of our study is in congruent with a previous study [8]. With regards to attitude towards preeclampsia, 120 (29.3 %) of the study participants had a positive attitude towards the risk factors, the prevention, signs, and symptoms of preeclampsia in this study. A similar finding was reported in a study conducted in Naples, Italy which stated only 21.7 % of the study participants were found to be worried about pregnancy risk factors [12].

The finding of our study indicated that the education level of the study participants was found to be significantly associated with the level of knowledge on preeclampsia. Participants who did not attend any formal education were less likely to be knowledgeable on preeclampsia compared with those who attended college and above level of education (AOR = 0.22, 95 % CI (0.06, 0.85)). The finding of this study is found to be supported by studies conducted in Ghana [25] and India [23]. The finding of this study publicized that improving female education is an important means of addressing reproductive health problems aside from fulfilling the international commitment of eliminating gender disparities in education at the end of 2030 which is targeted by the SDGs [15].

With regards to the timing of ANC visits, participants who visited health facilities for booking in the first trimester were 6.59 times more likely to have good knowledge on preeclampsia compared with those who visited health facilities for ANC booking in the third trimester (AOR = 6.59, 95 % CI (1.43, 30.33). Those who visited the health facilities for ANC booking in the first trimester have the opportunity to follow counseling about a healthy lifestyle such as a balanced diet, avoiding stress, and regular checkups in contrast to those who visited health facilities for ANC booking in the third trimester [23]. Moreover, those who visit health facilities for ANC booking in early gestation are likely to be educated so that they might have good knowledge on preeclampsia compared to their counterparts. Hence, encouraging early initiation of ANC visit is crucial to improve women’s knowledge on preeclampsia and other pregnancy danger signs which also provides the opportunity to implement health promotion interventions in the maternal continuum of care [27].

The number of ANC visits was found to be the predictor of having good knowledge of preeclampsia. Participants who attended only one ANC visit were less likely to have good knowledge on preeclampsia compared with those who attended four and more ANC visits (AOR = 0.13, 95 % CI (0.03, 0.59)). Those who had four and more ANC visits are likely to be counseled about pregnancy danger signs including preeclampsia causes, risk factors, and prevention mechanisms in every visit. Women who visit ANC clinics at appointment times are likely to be remained about danger signs and healthy diet frequently in contrast to those who had limited ANC visits [26, 28]. The finding of this study suggested that encouraging women to have four and more ANC visits is crucial to increase women’s knowledge on preeclampsia aside from enabling them to give birth at health facilities. The finding of our study is supported by a study in India [27].

The place of the last birth was found to be a determinant factor for being knowledgeable on preeclampsia. The odds of having good knowledge on preeclampsia were found to be 2.61 times higher among women who gave birth to their last child at a health institution compared with those who gave birth at home (AOR = 2.61, 95 % CI (1.03, 6.61)). The finding of this study implied that those who gave birth at health facilities in the previous births are likely to be counseled about preeclampsia including other danger signs in contrast to their counterparts [28].

Similarly, those who had experienced an obstetric complication in their previous births were 3.67 times more likely to have good knowledge on preeclampsia compared with their counterparts (AOR = 3.67, 95 % CI (1.78, 7.57)). This finding indicated that women who experienced a certain condition are more likely to be aware of the condition compared to counterparts. Women would seek prompt medical care when they are aware of the likely consequences of the symptoms they experience compared to those who did not have [25].

## Conclusions

No formal education and not attending four or more ANC visits were associated with poor knowledge of preeclampsia. While participants who visited health facilities during the first trimester, who gave birth at health facilities, and those who experienced a complication in previous births were more likely to be knowledgeable on preeclampsia. Improving the numbers of ANC visits and encouraging facility delivery are important measures to improve women’s knowledge on preeclampsia. Health education regarding preeclampsia risk factors, symptoms, and complications shall be emphasized at times of ANC visits.

## Data Availability

All relevant data are available in the manuscript and its Supporting Information files without restriction.

## Abbreviations

AZPH: Addis Zemen Primary Hospital
ANC: Antenatal Care
ETB: Ethiopian Birr
DTGH: Debre Tabor General Hospital
MEPH: Mekane Eyesus Primary Hospital
NMPH: Nefas Mewucha Primary Hospital
MDGs: Millennium Development Goals
SDGs: Sustainable Development Goals
USD: United States Dollar
VIF: Variance Inflation Factor
